# Emerging themes in idiopathic intracranial hypertension

**DOI:** 10.1007/s00415-020-10090-4

**Published:** 2020-07-22

**Authors:** Olivia Grech, Susan P. Mollan, Benjamin R. Wakerley, Zerin Alimajstorovic, Gareth G. Lavery, Alexandra J. Sinclair

**Affiliations:** 1 Metabolic Neurology, Institute of Metabolism and Systems Research, College of Medical and Dental Sciences, University of Birmingham, Birmingham, B15 2TT UK; 2 Centre for Endocrinology, Diabetes and Metabolism, Birmingham Health Partners, Birmingham, B15 2TH UK; 3 Birmingham Neuro-Ophthalmology, Ophthalmology Department, Queen Elizabeth Hospital, University Hospitals Birmingham NHS Foundation Trust, Birmingham, B15 2TH UK; 4 Department of Neurology, Gloucestershire Royal Hospital, Gloucester, UK; 5 Department of Neurology, Queen Elizabeth Hospital, University Hospitals Birmingham NHS Foundation Trust, Birmingham, B15 2TH UK

**Keywords:** Glucagon-like peptide-1 (GLP-1), Intracranial pressure, Idiopathic intracranial hypertension, Headache, Obesity, Papilloedema

## Abstract

**Purpose:**

Idiopathic intracranial hypertension (IIH) is a rare disorder characterised by raised intracranial pressure. The underlying pathophysiology is mostly unknown and effective treatment is an unmet clinical need in this disease. This review evaluates key emerging themes regarding disease characteristics, mechanisms contributing to raised intracranial pressure and advances in potential therapeutic targets.

**Findings:**

IIH is becoming more common, with the incidence rising in parallel with the global obesity epidemic. Current medical management remains centred around weight management, which is challenging. Metabolic investigations of patients have identified specific androgen profiles in cerebrospinal fluid (CSF), which suggest an endocrine dysfunction impacting CSF secretion in IIH. Glucagon-like peptide-1 (GLP-1) and 11β-hydroxysteroid dehydrogenase type 1 (11β-HSD1) have been found to play a role in CSF dynamics in IIH and have formed the basis of the first clinical trials looking at new treatments.

**Conclusions:**

Identification of novel molecular targets thought to underlie IIH pathology is now being translated to clinical trials.

## Background

Idiopathic intracranial hypertension (IIH), also known as pseudotumor cerebri, is a rare disorder of unknown cause characterised by raised intracranial pressure (ICP) in the absence of underlying structural pathology. IIH typically affects women of reproductive age, with a significant association with obesity and recent weight gain [1, 2]. Although IIH is considered a rare syndrome, the incidence is increasing [3], and appears to reflect country specific rates of obesity [4].

The majority of patients with IIH present with headache, visual disturbance (e.g. loss of peripheral vision, transient visual obscurations, horizontal double vision) and pulsatile tinnitus. Some patients also experience back pain, dizziness and cognitive disturbances [1, 5, 6]. Papilloedema (swelling of the optic nerve head) is a hallmark feature of IIH and left untreated could lead to optic atrophy and permanent visual loss [7]. Rarely, patients present with papilloedema in the absence of any other symptoms. Preserving the vision is a key priority in the management of IIH. Commonly, patients with IIH develop chronic headaches, which significantly reduce quality of life and are typically difficult to treat [8, 9].

In 2018, the first consensus guidelines for the investigation and management of IIH in adults was established [2]. It outlines diagnostic principles and key management strategies, namely to treat underlying disease accomplished by weight loss, preserve vision via emergency surgery (when required) and minimise headache morbidity.

The exact pathology underlying IIH remains unknown. Improved treatment options are an unmet clinical need in this disease area [10]. Recent mechanistic studies have identified therapeutic targets such as glucagon-like peptide-1 (GLP-1) [11] and 11β-hydroxysteroid dehydrogenase type 1 (11β-HSD1), which have formed the basis of IIH randomised clinical trials [12–14]. The aim of this review is to inform the reader of emerging issues and present new key highlights in the therapeutic development for IIH.

### Epidemiology

IIH is considered a rare condition. Previously, the incidence within the general population was believed to be between 0.5 and 2 per 100,000 [15–19]. Recent evaluations in the UK, including the largest cohort study to date, reported a 108% increase from 2.26 to 4.69 per 100,000 between 2002 and 2016 [3]. Another large case controlled cohort study found the incidence of IIH in females had tripled from 2.5 to 9.3 per 100,000 between 2005 and 2017 [20]. Secondary to the rising incidence of IIH is the health care cost of the disorder: annual hospital costs in England rose from £9.2 million to £49.9 millon between 2002 and 2014 and was predicted to rise to £462.7 million by 2030 [3]. In 2007 in the US, IIH patients had exceptionally high admission rates of 38% of all those coded with IIH for that year, with costs exceeding $444 million. This estimate also included lost work income [21]. Repeat hospital admissions may reflect that IIH is a disease of social deprivation [20], but also highlights the ineffectiveness of IIH treatment for decades.

Obesity (> 90%), female sex and reproductive age are significant risk factors associated with IIH [1]. An epidemiology study in the US found that women have an eight times likelihood of the disorder compared to men, [17] which is significantly increased when overweight and of reproductive age (20–44 years) with an incidence of 12–20 per 100,000 [15–17]. Paediatric studies of IIH are not well established; however, one UK study found an incidence of 0.71 per 100,000, which was found to increase with age. For children aged 12–15 years, there is also an association with obesity, whereas the pathophysiology in younger patients remains unclear [22]. IIH in males is uncommon; however, of note males are more likely to develop severe visual loss [23].

## Working towards understanding the mechanisms of raised ICP

The James Lind Alliance research priority setting partnership gave voice to patients and medical professionals to outline the most important topics regarding IIH. Identifying the underlying biological mechanisms was recognised as the most important topic [10]. Whilst it is thought that IIH is a multifactorial disorder, altered CSF dynamics is a final common pathway [6]. The choroid plexus (ChP) is the primary site of CSF production and secretion. It is composed of specialised epithelial cells which utilise the Na^+^/K^+^-ATPase ion pump to move Na^+^ across the apical membrane and create an osmotic gradient to regulate CSF movement [24, 25]. Targeting receptors and channels implicated in CSF production including aquaporins, transient receptor potential vanilloid type 4 (TRPV4), sodium potassium cotransporter (NKCCl) and GLUT1 poses potential treatments for CSF disorders [26]. It should be noted that CSF plays a role in protein and metabolite clearance in the CNS; therefore, modulating its production and movement may potentially have deleterious effects. There are several hypotheses, which all relate to disturbances of this CSF equilibrium.

### Glucagon-like peptide-1

Glucagon-like peptide-1 (GLP-1) is a gut peptide secreted in response to food by the distal small intestine and stimulates glucose-dependent insulin secretion [27]. It is also synthesised by neurons in the nucleus tractus solitarius and is involved in satiety and weight loss [28]. In the distal proximal tubule, it interacts with GLP-1 receptors (GLP-1R) to stimulate cAMP-dependent pKA pathways, which result in prevention of Na^+^ absorption into the bloodstream [29]. The ChP also express GLP-1R and interaction with its agonist exendin-4, was demonstrated to reduce Na^+^/K^+^ ATPase, a surrogate measurement of CSF secretion [11]. Single subcutaneous administration of exendin-4 in rat models of raised ICP were able to successfully lower ICP for 24 h, demonstrating the efficacy of this potential drug [11]. GLP-1R agonists are already licensed for the use in diabetes and obesity. The IIH Pressure Trial, ISRCTN12678718, is a randomised controlled trial assessing the effect of repurposing this drug in patients with raised ICP and the results from this trial should be released soon [14].

### Androgen excess

The predominant risk factors for IIH, including female sex, obesity and reproductive age, suggest a contribution of sex hormones in the pathophysiology of raised ICP. The prevalence of polycystic ovarian syndrome (PCOS) has been found higher in studies of IIH patients than the general population and the phenotypes of both disorders; namely female sex and obesity, are similar [30]. Androgen excess have been associated with IIH, with increased circulating levels of androgens associated with earlier onset in women [31], and female-to-male transgender patients developing IIH after commencing testosterone therapy [32]. Using liquid chromatography–tandem mass spectrometry (LCMS), it has been possible to define a metabolome signature in the serum and CSF of IIH patients, which features excess androgens but is distinct from obesity and PCOS [33]. Until recently, the role of excess testosterone in IIH pathology was unknown. Treatment of rat ChP tissue with testosterone exhibited a marked increase in Na^+^/K^+^ ATPase activity, a surrogate measurement of CSF secretion. This in combination with the finding of androgen receptors on the human ChP, provides evidence for the role of androgen excess in increased CSF secretion [33].

### 11β-Hydroxysteroid dehydrogenase type 1

The enzyme 11β-hydroxysteroid dehydrogenase type 1 (11β-HSD1) has a key role in regulating CSF secretion by converting cortisone to its active form cortisol, which amplifies glucocorticoid signalling pathways and facilitates the transport of Na^+^ ions. It is expressed and active in the ChP and has demonstrated a role in IIH pathology [34]. 11β-HSD1 offers a link between obesity and raised ICP, as global activity was found to be reduced following therapeutic weight loss in IIH patients, which also correlated with a reduction in ICP [35]. The enzyme has been previously found to be dysregulated in obesity [36], with high levels found in human fat [37] and overexpression mouse models resulting in visceral obesity and a metabolic disorder [38]. Phenotyping studies have begun to elucidate a metabolically distinct profile of adipose tissue of IIH patients. LCMS-based 11β-HSD1 assays in the adipose tissue of IIH patients demonstrated an increased generation of cortisol when treated with cortisone, despite no differences in 11β-HSD1 gene expression [39].

Selective inhibitors of 11β-HSD1 have been used to treat obesity, a metabolic syndrome and diabetes mellitus type 2 [40]; therefore, the potential for these to reduce ICP has also been hypothesised. Recently a double-blind randomized controlled trial in the UK was able to demonstrate that AZD4017, a 11β-HSD1 inhibitor reduced ICP in IIH patients which was correlated to a reduction in serum cortisol:cortisone ratio [12]. This was the first phase II randomized trial of any medicine in IIH and confirmed the safety and tolerability of this 11β-HSD1 inhibitor.

### Cytokines and adipokines

Obesity is a chronic inflammatory condition, in which adipose tissue is capable of functioning as an endocrine organ, secreting a number of pro-inflammatory factors including cytokines, adipokines and chemokines [41]. As cytokine expression profiles are significantly different in IIH patients, could these factors have a role in IIH? [42]. Studies utilising miRNA/mRNA analysis have highlighted abnormalities in pro-inflammatory pathways in the CSF and serum of patients with raised ICP [43]. In particular, compared to controls, chemokine (C–C motif)-ligand 2 (CCL2) has been found to be significantly higher in the CSF of IIH patients, [42] whilst others found IL-2 and IL-17 to be significantly elevated [44]. These suggest a possible inflammatory pathway in IIH pathology and more work is underway to determine the significance of these findings, independent of the metabolic effects of obesity [42]. In one such experiment in the lab, one group of female rats was fed a high fat diet and another group were exposed to IIH-associated inflammatory factors. Both groups of animals exhibited increased CSF secretion with reduced CSF drainage when treated with CCL2 [45]. These studies further highlight a pathogenic link between weight gain and raised ICP. Could this pathway be targeted for a biomarker of altered CSF drainage, or as a therapy?

## Current medical management in IIH

**Fig. 1:**
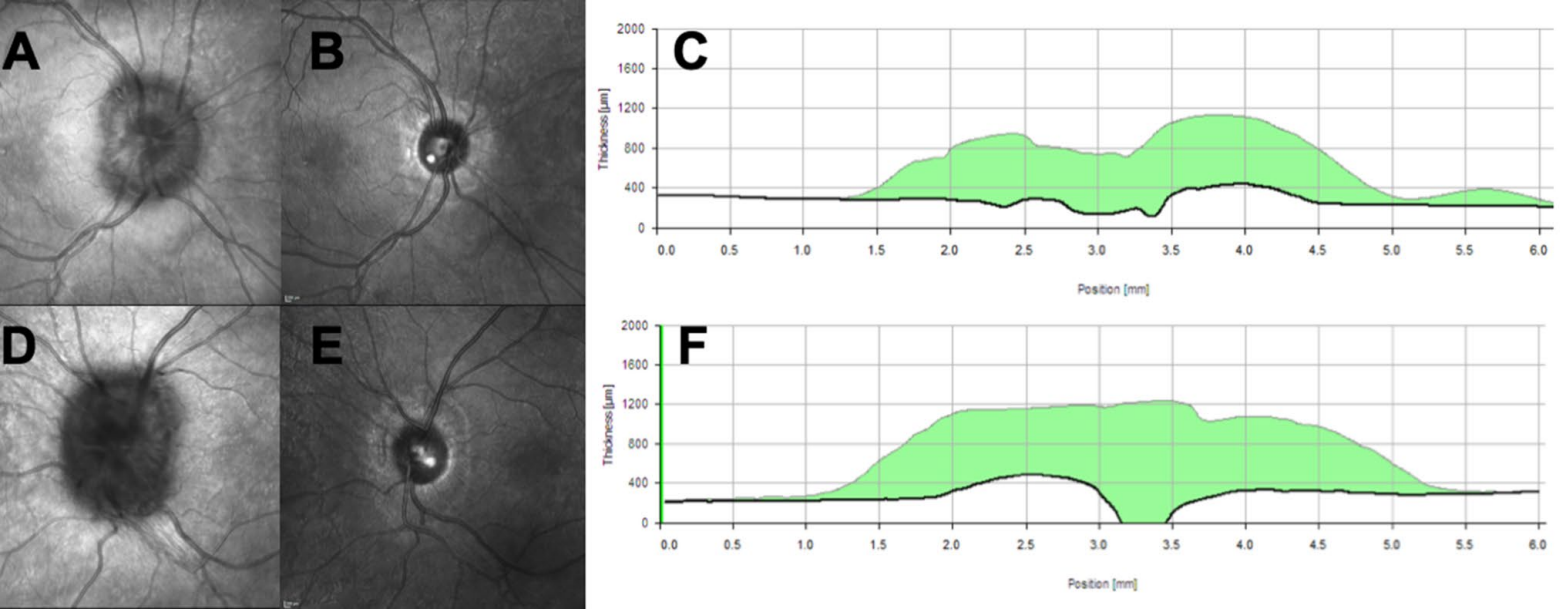
Optical coherence tomography (OCT) images from a young Caucasian woman who presented with papilloedema. CT head and CT venogram showed no abnormalities. CSF opening pressure was 52 cm CSF, with normal contents. Her weight was 99 kg and body mass index 40.2 kg/m^2^. Following lifestyle intervention of a calorie-controlled diet and exercise alone, she lost 15 kg (BMI 34.1) putting her disease into complete remission within 8 weeks. **a** Infrared image of the right optic nerve; **b** infrared image of the right optic nerve following weight loss; **c** shows the central cross section, and the amount of reduction in the retinal nerve fibre layer in the right eye over the 8 weeks. **d** Infrared image of the left optic nerve; **e** infrared image of the left optic nerve following weight loss; **f** shows the central cross section and the amount of reduction in the retinal nerve fibre layer in the left eye over the 8 weeks

Weight loss is an effective management strategy to induce IIH remission (Fig. 1). The Birmingham weight loss prospective trial [46] demonstrated that a very low-calorie diet (1777 kJ/day (425 kcal/day)) resulted in significant weight loss (15.3 ± 7.0% of body weight), significantly lowered ICP and led to a significant improvement in papilloedema, vision and headache outcomes. There are a number of case series detailing alternative methods of weight loss in IIH [47]. The results of one multicentre, randomised controlled trial designed to assess if weight loss through bariatric surgery is a more effective sustainable treatment for IIH than lifestyle modification through a community weight management program is currently awaited [48]. The IIH consensus guidelines recommend weight loss for all those who have typical IIH [2].

Acetazolamide is a carbonic anhydrase inhibitor widely used to lower ICP and is a first-line treatment in IIH. Although the exact mechanism of action of acetazolamide on CSF dynamics remains unknown, cell models have demonstrated that it inhibits Na^+^/K^+^ ATPase in the ChP [49, 50]. The IIH treatment trial (IIHTT) was a multicentre randomised, double-blind study, which investigated the use of acetazolamide in conjunction with a low sodium diet in IIH patients with mild visual loss and reported modest improvement in the perimetric mean deviation of the visual field [51]. In clinical practice, acetazolamide is poorly tolerated by many patients [51, 52], and more recently the clinical benefit of acetazolamide has been questioned [53]. One preclinical study reported that acetazolamide had no effect on ICP in female rats and that topiramate in comparison was far superior at reducing ICP [54]. Topiramate is also a carbonic anhydrase inhibitor and has the additional benefits of inducing weight loss in some patients and being an effective migraine preventative [55]. Although Topiramate has been reported to relieve symptoms in IIH [56, 57], further controlled trials with it are required to see whether it is truly effective in treating this condition.

## Headache in IIH

### Clinical features

There are few clinical studies characterising IIH headache [9]. Previously, IIH headaches were considered “high pressure” and, therefore, aggravated by Valsalva manoeuvre, worse when supine and often present on waking or causing the patient to wake from sleep [9, 58]. However, more recent studies indicate that the majority of headaches in IIH meet the International Headache Society criteria for either episodic migraine, chronic migraine or tension-type headache [51, 59, 60]. In the IIHTT, 84% of participants had headache at baseline [60]. Headache is a chronic disabler in IIH and significantly reduces quality of life [8]. The relationship between ICP and headache in IIH remains complicated. Although reduction in ICP was found to alleviate headache in weight loss studies [46], severity, frequency and disability of headache did not correlate with lumbar puncture opening pressure at baseline in the IIHTT [60].

### Management

The phenotype of IIH headache appears to mirror that of episodic and chronic migraine [9] and increasingly off-label migraine treatment is used in IIH patients to treat headache without any formal evidence of efficacy [2]. Overuse of simple analgesics, opiates, and non-steroidal anti-inflammatory drugs is common in IIH and may result in medication-overuse headache [61, 62]. Patients who achieve 10–15% weight loss often see an improvement in their headache [46], although sometimes this can only be achieved with bariatric surgery [63]. Although therapeutic LP may reduce headache in the short term, repeated LPs are not recommended as they may result in complications such as intracranial hypotension [64, 65]. Headache generation and pain are thought to be due to peripheral sensitisation of the trigeminovascular system, in which innervation of the dura by nociceptive trigeminal fibers, leads to release of vasoactive neuropeptides including calcitonin gene-related peptide (CGRP) and substance P [66–68]. In the case of migraine, repeated periods of sensitisation over time is thought to cause a decrease in nociceptive threshold and may result in chronicity [69]. CGRP is thought to play an important role in the pathogenesis of migraine. Plasma CGRP is elevated during migraine attacks and administration of exogenous CGRP may induce migraine without aura in sufferers. Recent trials also demonstrate that monoclonal antibodies against CGRP or the CGRP receptor are effective for the treatment of chronic and episodic migraine [59, 70–75]. Evidence of CGRP involvement in post-traumatic headache, [76, 77] which also features raised ICP, suggests that these therapeutics would also be effective for IIH headache. Furthermore, the headache phenotype in IIH is typically migraine [60]. At present, there is no trial evidence for the use of CGRP monoclonal antibodies to treat headaches with a migrainous phenotype in IIH.

## Vision in IIH

### Clinical features

Visual symptoms of raised intracranial pressure may include: visual blurring, transient visual obscurations (TVOs) and double vision [7]. Often patients can have a mild hyperopic shift due to papilloedema causing visual blurring. TVOs, where there is a short-lived greying or blacking out of the vision in either or both eyes, with return to normal vision, is more common in the acute setting of raised ICP. These often happen on bending or during a Valsalva manoeuvre. Where they increase in frequency at rest is a red flag of progression to fulminant disease requiring urgent assessment. If horizontal binocular diplopia is reported, then a full extra ocular movement examination will likely reveal a unilateral or bilateral sixth-nerve palsy [7]. Rarely other cranial nerve palsies have been reported in IIH [2]. If monocular diplopia is reported, a close examination of the macula with fundoscopy and OCT may reveal either fluid in the acute setting or an epiretinal membrane in the chronic setting.

### Examination

On examination papilloedema (unilateral or bilateral disc swelling) is one of the essential features required to diagnose IIH [78]. However, examination of the fundus can be challenging [58], and up to 40% of those sent to a tertiary centre had an incorrect diagnosis of IIH made due to diagnostic error in the fundal examination [79]. If there is any clinical uncertainty, papilloedema should be confirmed by an experienced specialist, [2] as optic disc drusen, small hypermetropic discs, titled myopic discs and vitreous traction can all be mistaken for papilloedema [7].

**Fig. 2:**
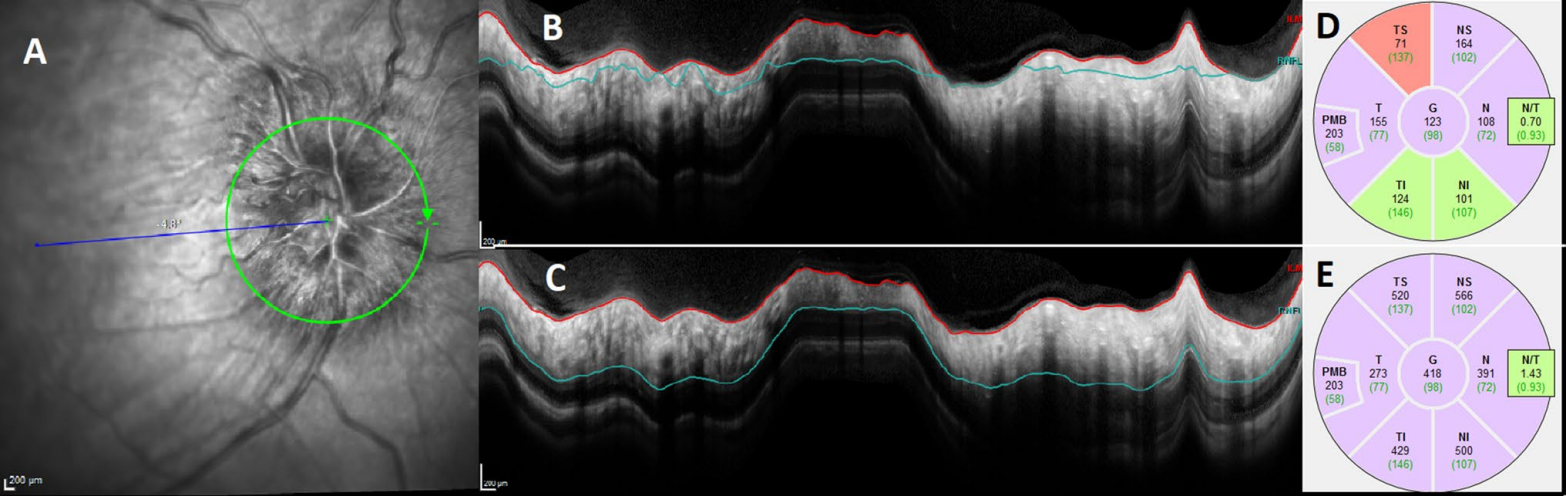
Segmentation error is common in higher grades of papilloedema. **a** The infrared image of the right eye with a Frisén grade 3 disc swelling, as graded on biomicroscopy. **b** The blue line, segmenting the retinal nerve fibre layer (RNFL), has been automatically placed in the incorrect area. **c** the manual resegmentation of the RNFL. **d** The initial figures for the retinal nerve fibre layer values in each segment. **e** The resegmented values of the RNFL, and can be compared directly to **d**, highlighting the difference that might clearly change clinical management when longitudinally following a patient

Testing visual function is essential as not only is there no correlation between headache frequency and the degree of papilloedema [80], there is little correlation between papilloedema grade and LP OP. Visual function importantly guides management, particularly in the acute setting of diagnosis or in established IIH with an acute exacerbation of headache [81]. The minimum visual data set recommended is a visual acuity, pupil examination, formal visual field assessment and dilated fundal examination [2, 82]. Where possible ocular imaging is helpful at baseline to document papilloedema and is essential for longitudinal follow-up either by photography or optical coherence tomography (OCT) (Fig. 1). Indeed certain OCT measures show the ability to help distinguish between papilloedema and pseudopapilloedema [83] and other investigators have found associations and correlations with ICP [84]. As with any device, care in interpretation is important due to proprietary software errors in moderate to severe papilloedema (Fig. 2) [85].

Visual fields are typically the first part of the visual function affected by ICP with peripheral constriction, an enlargement of the blind spot and or an inferior nasal step or partial arcuate defect [86, 87]. For the general neurologist, interpretation of the visual fields is essential as in IIH cognitive factors including subject attention, motivation, fatigue, and response bias can influence the results. For example, in the IIH TT, one-fifth of the visual fields had to be repeated due to poor reliability [88]. Understanding of the type of visual field loss and how to interpret the plots presented, reliability indices and global parameters is helpful [82].

## Emergency management

Less than 10% of those with IIH [3] present with rapidly progressive loss of visual function (termed fulminant IIH) and in whom an acute reduction in ICP is required to preserve vision, and so in these cases, surgical intervention is necessary [2, 82]. Established options include CSF diversion with the commonest surgeries being a ventriculoperitoneal (VP) shunt or lumboperitoneal (LP) shunt, or optic nerve sheath fenestration (ONSF). CSF diversion and shunt revision surgeries are the commonest intervention for fulminant IIH [3], with a third of patients requiring multiple revision surgeries [89]. Ventriculoperitoneal shunts are currently preferred [2, 82], due to the reported lower revision rates compared to lumboperitoneal shunts (1.8 versus 4.3 revisions per patient, respectively [90]).

More recently cerebral venous sinus stenting (CVSS) has been popularized through various case series and may be of particular benefit in these urgent cases [91, 92]. Saber et al. concluded that in patients with refractory IIH and venous sinus stenosis with elevated pressure gradients, venous sinus stenting was associated with a reduction in pressure gradient and ICP, improvement in signs and symptoms of IIH and acceptable stent survival rates [92]. The rate of serious complications (including intracranial haemorrhage, venous sinus thrombosis and ultimately death) has been reported as less than 2% in a number of systematic reviews [93], and around 5% in a large case series [94].

There is a lack of randomised controlled trials (RCT) evaluating the effectiveness of all the surgical interventions for IIH and the effectiveness of each remains uncertain. Comparing the three interventions, Satti et al. reported that the overall rate of serious complication following venous sinus stenting (2.9%) was higher than following OSNF (1.5%) but significantly lower than following CSF diversion (7.6%) [95].

## Conclusion

Basic science research has started to uncover the metabolic aetiology of IIH. There is a clear unmet need for biomarkers of disease activity and better tolerated treatments. Translating this knowledge will better serve our patients and health community, where IIH still carries a significant stigma and bias due to its association with obesity.
